# Satratoxin G from the Black Mold *Stachybotrys chartarum* Evokes Olfactory Sensory Neuron Loss and Inflammation in the Murine Nose and Brain

**DOI:** 10.1289/ehp.8854

**Published:** 2006-02-27

**Authors:** Zahidul Islam, Jack R. Harkema, James J. Pestka

**Affiliations:** 1 Center for Integrative Toxicology; 2 Department of Microbiology and Molecular Genetics; 3 Department of Food Science and Human Nutrition and; 4 Department of Pathobiology and Diagnostic Investigation, Michigan State University, East Lansing, Michigan, USA

**Keywords:** apoptosis, fungus, inflammation, inhalation, mycotoxin, neurotoxicity, olfactory sensory neuron, rhinitis, trichothecene

## Abstract

Satratoxin G (SG) is a macrocyclic trichothecene mycotoxin produced by *Stachybotrys chartarum*, the “black mold” suggested to contribute etiologically to illnesses associated with water-damaged buildings. Using an intranasal instillation model in mice, we found that acute SG exposure specifically induced apoptosis of olfactory sensory neurons (OSNs) in the olfactory epithelium. Dose–response analysis revealed that the no-effect and lowest-effect levels at 24 hr postinstillation (PI) were 5 and 25 μg/kg body weight (bw) SG, respectively, with severity increasing with dose. Apoptosis of OSNs was identified using immunohistochemistry for caspase-3 expression, electron microscopy for ultrastructural cellular morphology, and real-time polymerase chain reaction for elevated expression of the proapoptotic genes *Fas*, *FasL*, *p75NGFR*, *p53*, *Bax*, caspase-3, and *CAD*. Time-course studies with a single instillation of SG (500 μg/kg bw) indicated that maximum atrophy of the olfactory epithelium occurred at 3 days PI. Exposure to lower doses (100 μg/kg bw) for 5 consecutive days resulted in similar atrophy and apoptosis, suggesting that in the short term, these effects are cumulative. SG also induced an acute, neutrophilic rhinitis as early as 24 hr PI. Elevated mRNA expression for the proinflammatory cytokines tumor necrosis factor-α, interleukin-6 (IL-6), and IL-1 and the chemokine macrophage-inflammatory protein-2 (MIP-2) were detected at 24 hr PI in both the ethmoid turbinates of the nasal airways and the adjacent olfactory bulb of the brain. Marked atrophy of the olfactory nerve and glomerular layers of the olfactory bulb was also detectable by 7 days PI along with mild neutrophilic encephalitis. These findings suggest that neurotoxicity and inflammation within the nose and brain are potential adverse health effects of exposure to satratoxins and *Stachybotrys* in the indoor air of water-damaged buildings.

Numerous adverse human health effects have been attributed to damp indoor air environments generated by aberrant water exposure due to excessive condensation and failure of water-use devices, as well as building envelope breach during heavy rains or flooding, as occurred during Hurricanes Katrina and Rita on the Gulf Coast of the United States. An Institute of Medicine (IOM) expert panel concluded that an association exists between damp buildings and upper respiratory tract symptoms, wheeze, cough, and exacerbation of chronic lung diseases such as asthma, whereas supportive data for other reported outcomes such as neurocognitive dysfunction, mucous membrane irritation, fatigue, fever, and immune disorders are lacking (IOM 2004). Building-related illnesses are often linked to dampness-promoted growth of fungi (Fog Nielsen 2003) and, most notably, *Stachybotrys chartarum*, a saprophytic “black mold” that grows on cellulosic materials, including wall-board, ceiling tiles, and cardboard (Hossain et al. 2004). Incidences of indoor *S. chartarum* contamination often generate costly litigation and remediation, are extensively reported by the media, and have evoked intense public and scientific controversy (Hardin et al. 2003). The IOM panel suggested that although *in vitro* and *in vivo* research on *S. chartarum* and its mycotoxins suggests that adverse effects in humans are indeed “biologically plausible,” their association with building-related illnesses requires rigorous validation from the perspectives of mechanisms, dose response, and exposure assessment (IOM 2004).

The satratoxins, macrocyclic trichothecenes produced by *S. chartarum*, are potent inhibitors of protein translation that initiate both inflammatory gene expression and apoptosis *in vitro* after upstream activation of mitogen-activated protein kinases (MAPKs) (Chung et al. 2003; Yang et al. 2000). Satratoxin equivalent airborne concentrations ranging from 2 to 34 ng/m^3^ (Yike et al. 1999) and from 54 to 330 ng/m^3^ (Vesper et al. 2000) have been previously estimated, by a translational bioassay, to occur in rooms of water-damaged homes heavily contaminated with *Stachybotrys*. These water-soluble mycotoxins occur in the outer plasmalemma surface and the inner wall layers of conidiospores (Gregory et al. 2004) as well as in nonviable airborne particulates (Brasel et al. 2005), which could facilitate entry and release into respiratory airway tissue. Indeed, pulmonary toxicity of the spores of *S. chartarum* and associated trichothecenes has been demonstrated in animal studies using intranasally or intratracheally exposed laboratory rodents (Yike and Dearborn 2004; Yike et al. 2005).

Under normal resting, nonexercising conditions, the nose functions to filter, warm, and humidify inhaled air before it enters more delicate airway and alveolar tissues in the distal lung (Cole 1993). Nasal passages serve as “scrubbing towers” for the respiratory tract by efficiently *a*) absorbing water-soluble and reactive gases and vapors, *b*) trapping inhaled particles, and *c*) metabolizing airborne xenobiotics (Harkema 1991). Given these air conditioning and defensive roles, we hypothesized that the nasal airways are another critical site for interaction with *S. chartarum* mycotoxins. To test this hypothesis, we employed a murine intranasal instillation model previously used by our laboratory and others to study the adverse effects of harmful toxic agents (Giannetti et al. 2004), allergens (Farraj et al. 2004), and pathogens (Wiley et al. 2001) to investigate potential nasal toxicity of satratoxin G (SG), one of the most potent trichothecenes produced by *S. chartarum* (Yang et al. 2000).

## Materials and Methods

### Toxins

SG and isosatratoxin F (ISF) were purified from *S. chartarum* cultures and kindly provided by B. Jarvis (University of Maryland, College Park, MD). SG and ISF yielded a single peak at 254 nm by the HPLC method of Hinkley and Jarvis (2001). SG and ISF identities were further confirmed by electrospray ionization/collision-induced dissociation (ESI-CID) tandem mass spectroscopy at the Michigan Sate University mass spectrometry facility by a modification of a published method (Tuomi et al. 1998) using a LCQ-DECA device (Finnigan, San Jose, CA) fitted with an ESI probe. Deoxynivalenol, T-2 toxin, and verrucarin A (Sigma Chemical Co., St. Louis, MO) had reported purities of > 98%, 98%, and 95%, respectively.

### Laboratory animals and intranasal instillation

Mice were maintained under humane conditions according to National Institutes of Health guidelines (Institute of Laboratory Animal Resources 1996) as overseen by the All University Committee on Animal Use and Care at Michigan State University. Pathogen-free female C57Bl/6 mice (7–8 weeks of age; Charles River, Portage, MI) were randomly assigned to experimental groups (*n* = 5–6) and housed in polycarbonate cages containing Cell-Sorb Plus bedding (A & W Products, Cincinnati, OH) covered with filter bonnets and provided free access to food and water. Room lights were set on a 12-hr light/dark cycle, and temperature and relative humidity were maintained between 21 and 24°C and 40 and 55%, respectively. For each experiment, mice were anesthetized with 4% halothane and 96% oxygen and then instilled intranasally at 50 μL/mouse with SG or other trichothecenes dissolved in a vehicle of pyrogen-free saline (Abbott Laboratories, Abbott Park, IL) or with the vehicle alone.

### Animal necropsies and tissue processing for light microscopic examination

For histopathology and morphometry, mice were deeply anesthetized via intraperitoneal (ip) injection of 0.1 mL of 12% sodium pentobarbital in saline at designated times post-instillation (PI), from 6 hr to 28 days, and killed via exsanguination by cutting the abdominal aorta or renal arteries. Heads from each mouse were immediately removed, and 1 mL of 10% neutral buffered formalin (Fisher Scientific Co., Fairlawn, NJ) was flushed retrograde through the nasopharyngeal meatus. After the lower jaw, skin, muscles, eyes, and dorsal cranium were removed, the head with brain intact was immersed and stored in a large volume of the fixative for at least 48 hr before further tissue processing. Lungs were also removed and intratracheally perfused with formalin fixative at a constant pressure of 30 cm of water for approximately 1 hr and then similarly immersed and stored for a minimum 48 hr.

**Figure 1:**
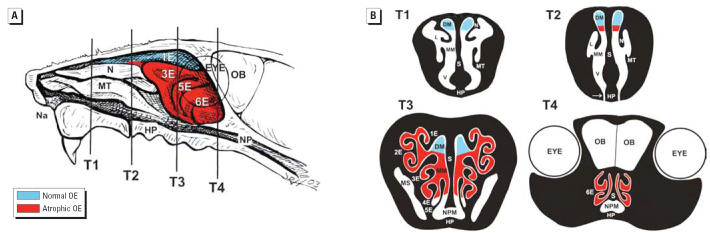
Diagrammatic representation of the nasal passages of the murine nose. Abbreviations: DM, dorsal medial meatus (airway); E, ethmoid turbinate; HP, hard palate; L, lateral meatus; MM, middle meatus; MS, maxillary sinus; MT, maxilloturbinate; N, nasoturbinate; Na, naris; NP, nasopharynx; NPM, nasopharyngeal meatus; S, septum; V, ventral meatus. (*A*) Right nasal passage with the nasal septum removed, exposing the luminal surfaces of nasal turbinates (N, MT, 1E–6E) projecting from the lateral wall; vertical lines indicate the anterior surfaces of transverse tissue blocks (T1–T4) that were selected for microscopic examination. (*B*) Cross-sectional views of T1–T4. Colored areas in (*A*) and (*B*) represent OE that exhibited SG-induced apoptosis and atrophy 1–7 days PI or were free of toxin-induced injury.

After fixation, transverse tissue blocks from the head and left lung lobe of these mice were selected for light microscopy as previously described (Steiger et al. 1995). Before sectioning, the heads were decalcified in 13% formic acid for 7 days and then rinsed in tap water for at least 4 hr. The nasal cavity of each mouse was transversely sectioned at four specific anatomic locations, designated T1–T4 (Mery et al. 1994; Young 1981). The most proximal nasal section was taken immediately posterior to the upper incisor teeth (proximal, T1); the middle section was taken at the level of the incisive papilla of the hard palate (middle, T2); the third nasal section was taken at the level of the second palatal ridge (T3); and the most distal nasal section (T4) was taken at the level of the intersection of the hard and soft palate and through the proximal portion of the olfactory bulb (OB) of the brain (Figure 1). In addition, two transverse tissue blocks from the left lung lobe were also taken for microscopic examination at the level of airway generation 5 (proximal) and generation 11 (distal) along the main axial airway. Tissue blocks were embedded in paraffin, and the anterior face of each block was sectioned at a thickness of 5 μm, and stained with hematoxylin and eosin (H&E).

### Immunohistochemistry

Unstained and hydrated paraffin sections from nasal blocks T3 and T4 were incubated first with a nonspecific protein-blocking solution containing normal sera (Vector Laboratories Inc., Burlingame, CA) and then with specific dilutions of primary polyclonal antibodies directed against activated caspase-3 (1:100, rabbit anti-caspase-3 antibody; Abcam, Inc., Cambridge, MA), olfactory marker protein (OMP; 1:4,000, goat anti-OMP antibody, provided by F. Margolis, University of Maryland), or infiltrating neutrophils (1:600, rabbit anti-rat neutrophil antibody, provided by R. Roth, Michigan State University). Tissue sections used for caspase-3 or OMP detection were pretreated before the blocking solution with 3% hydrogen peroxide in methanol to destroy endogenous peroxidase. With these tissue sections, the primary antibody was followed by the secondary antibody, biotinylated anti-species IgG. Immunoreactivity of caspase-3 and OMP was visualized with Vector R.T.U. Elite ABC-Peroxidase Reagent followed by Nova Red (Vector Laboratories Inc.) as the chromagen. Anti-neutrophil antibody treatment was followed by biotinylated anti-rabbit IgG, and then streptavidin-phosphatase complex (KPL laboratories, Gaithersburg, MD) and Vector red as the chromagen. After immunohistochemistry, slides were lightly counterstained with hematoxylin.

### Semiquantitative scoring of nasal histopathology

Nasal sections from mice that received a single instillation of SG at various doses and were sacrificed 24 hr PI were scored for the amount of toxin-induced, light microscopic lesions in the olfactory epithelium (OE). A veterinary pathologist, without previous knowledge of exposure history of the individual mice, ranked severity of SG-induced OE apoptosis with atrophy in the examined nasal tissue sections (T1–T4) using the following histopathologic numeric scores: 0, no SG-induced nasal lesions in OE; 1 (minimal), 25% of OE with lesions; 2 (mild), 25–50% of OE with lesions; 3 (moderate), 50–75% of OE with lesions; or 4 (marked), ≥ 75% of OE with lesions.

### Light microscopic morphometry

Thickness of the OE lining the medial surface of the second ethmoid turbinates (2E) in T3 (Figure 1) was morphometrically evaluated as previously described for airway epithelium (Hyde et al. 1990, 1991; Plopper et al. 1994). Measurements were conducted at a final magnification of 3,540× using a light microscope (Olympus BX40; Olympus America Inc., Melville, NY) coupled to a 3.3-megapixel digital color camera (Q-Color 3 Camera; Quantitative Imaging Corp., Burnaby, British Columbia, Canada), and a personal computer (Dimension 8200; Dell, Austin, TX). The morphometric analyses were performed using a cycloid grid overlay and software for counting points and intercepts (Stereology Toolbox; Morphometrix, Davis, CA) (Hyde et al. 1990, 1991). The percentage volume density, *V_v_*, the proportion of the epithelium composed of cytoplasm, nuclei, or apoptotic nuclear fragments, was determined by point counting and calculated using the following formula:


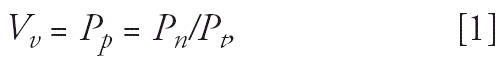


where *P_p_* is the point fraction of *P_n_*, the number of test points hitting the structure of interest, divided by *P_t_*, the total points hitting the reference space (OE). The volume of the epithelial component of interest (e.g., apoptotic nuclei) per unit of basement membrane (*S_v_*) was determined by point and intercept counting and was calculated using the following formula:


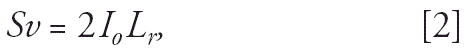


where *I_o_* is the number of intercepts with the object (epithelial basal lamina) and *L_r_* is the length of test line in the reference volume (epithelium). To determine thickness of the OE, a volume per unit area of basal lamina (cubic micrometers per square micrometer) was then calculated using the following formula for arithmetic mean thickness (τ):


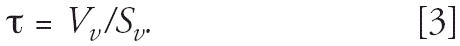


Other standard morphometric and image analysis techniques were used to determine the numeric cell density of mature olfactory sensory neurons (OSNs) in OE. Morphometric estimates of the numeric cell density of OSNs immunohistochemically reactive for OMP (protein indicator of mature OSNs) were determined via light microscopy (790× final magnification) by counting the number of nuclear profiles of these immunoreactive neuroepithelial cells in the OE lining the medial surface of 2E in T3 (Figure 1) and dividing by the length of the underlying basal lamina. The length of the basal lamina was determined from the contour length of a computerized digital image of the basal lamina using the Scion Image program (Scion Corporation, Frederick, MD). All numeric cell density data were expressed as the number of OSN nuclei per millimeter of basal lamina.

### Ultrastructural examination of the olfactory mucosa and OB via transmission electron microscopy

Mice designated for transmission electron microscopy (TEM) analysis were anesthetized with an ip injection of 0.1 mL 12% pentobarbital containing 1 IU heparin. Immediately after anesthesia, the whole body received an intravascular perfusion via the left heart with a saline solution containing 10 IU heparin for 2–3 min, followed by a 7–10 min perfusion with 4% glutaraldehyde fixative solution (Ted Pella, Inc., Redding, CA). The nasal cavity and brain were then removed and stored in the fixative until TEM processing after the nasal cavity was decalcified with 10% EDTA for 3–4 weeks; selected tissues from ethmoid turbinates and OB were postfixed in 1% phosphate-buffered osmium tetroxide, dehydrated through a graded series of ethanol and propylene oxide, and embedded in Poly/Bed-Araldite resin (Polysciences, Inc., Warrington, PA). Sections (1 μm) were cut and stained with toluidine blue for light microscopic identification of tissue sites for TEM. Ultrathin tissue sections for TEM were cut at approximately 75 nm with a diamond knife, mounted on copper grids, and stained with lead citrate and uranyl acetate. Sectioning was done with an LKB Ultratome III (LKB Instruments, Inc., Rockville, MD). Ultrastructural tissue examination and photography were performed with a JEOL JEM 100CXII electron microscope (JEOL Ltd., Tokyo, Japan).

### Real-time polymerase chain reaction

Mice used for polymerase chain reaction (PCR) analyses of nasal and brain tissues were anesthetized and killed at designated times after SG instillation as described above. Immediately after death, the head of each mouse was removed from the carcass; after the skin, muscles, eyes, and lower jaw were removed from the head, the nasal airways were opened by splitting the nose in a sagittal plane adjacent to the midline. The nasal septum was removed, thereby exposing the nasal turbinates projecting from the lateral wall of each nasal passage (Figure 1). Using a dissecting microscope and ophthalmic surgical instruments, all ethmoid turbinates and the OB were dissected from both nasal passages and brain, respectively. These excised tissues were stored in RNAlater (Ambion Inc., Austin, TX) within 5 min, and RNA was isolated using RNeasy Protect Mini kit (Qiagen Inc., Valencia, CA) within 7 days. Absence of RNA degradation was routinely verified by agarose electrophoresis; real-time PCR for apoptosis-related genes [*Fas*, *FasL*, p75-nerve growth factor receptor (*p75NGFR*), *p53*, *Bax*, *Bcl-2*, *Apaf-1*, caspase-3, and caspase-activated DNase (*CAD*)], cytokine genes [interleukin-1 (*IL-1*), tumor necrosis factor-α (*TNF-*α), *IL-6*], and the chemokine gene *MIP-2* (macrophage-inflammatory protein-2) were performed on an ABI PRISM 7900HT Sequence Detection System using Taqman One-Step RT-PCR (reverse-transcriptase-PCR) Master Mix and Assays-on-Demand primer/probe gene expression products according to the manufacturer’s protocols (Applied Biosystems, Foster City, CA). Relative quantification of apoptotic and cytokine gene expression was carried out using an 18S RNA as the loading control and an arithmetic formula method (Audige et al. 2003).

### Statistics

All data were analyzed with SigmaStat (version 3.1; Jandel Scientific, San Rafael, CA) with the criterion for significance set at *p* < 0.05. Morphometric and RT-PCR data were statistically analyzed using one-way analysis of variance with Student-Newman-Keuls posttest. Data from histopathologic severity scores of SG-induced lesions were analyzed using the Mann Whitney rank sum test (nonparametric test) with Bonferroni correction for multiple comparisons.

## Results

### OE targeted by nasal SG exposure

Light microscopic evaluation of four specific anatomical sites (T1–T4) revealed that mice exposed to SG [500 μg/kg body weight (bw)] and sacrificed at 1, 3, or 7 days PI had conspicuous nasal epithelial and inflammatory lesions in the dorsocaudal half of the nasal passages that is normally lined by OE. These lesions were not apparent in the nasal cavity of saline vehicle–treated controls (Figure 1A). SG-related alterations were neither present in regions of the nasal airways lined by other nasal epithelial types, including respiratory, transitional, or squamous epithelium, nor found in the lungs of exposed mice. In addition, mice that were sacrificed only 6 hr PI had no exposure-related lesions in their nasal cavities. SG-induced OE lesions at 1 day PI consisted of numerous individual epithelial cells with morphologic features characteristic of apoptosis in the OE lining all the ethmoid turbinates and the adjacent lateral walls that border the lateral meatus in the distal regions of both nasal passages (T3 and T4), with the most dorsolateral ethmoid turbinates (1E and 2E) being most severely affected (Figure 1B). SG-induced apoptosis was also present in the OE of the mid and ventral septum lining the middle medial meatus in the distal nasal passages (T3 and T4). In the middle of the nasal passages (T2), before the distal regions containing the ethmoid turbinates (T3 and T4), SG-induced apoptotic lesions in the OE were detected only in a small mucosal region of the lateral walls and septum lining the middle medial meatus where the OE meets the respiratory epithelium. SG-induced nasal epithelial lesions were undetectable in the most proximal regions of the nasal passages (T1). Interestingly, the OE lining the dorsal medial meatus throughout the nasal passages (T1–T4) had no microscopic evidence of SG-induced apoptosis or any other epithelial alterations.

### Apoptosis induction in OE

**Figure 2:**
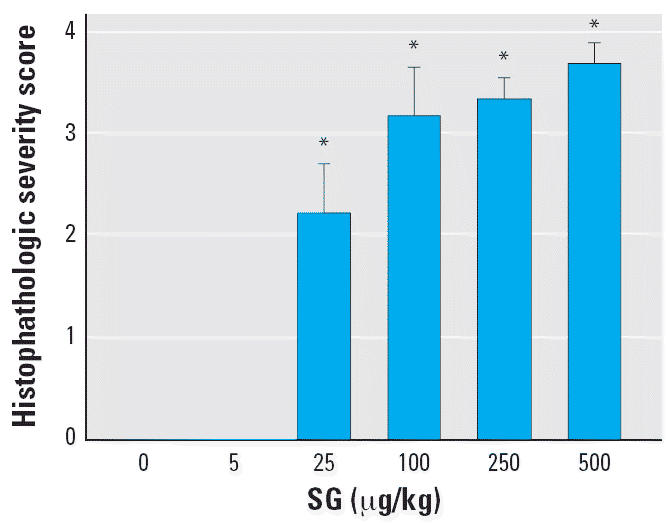
Effect of SG dose on OE toxicity shown as histologic severity score in nasal airways of treated mice (see “Materials and Methods” for details). Bars represent group means + SEs (*n* = 5–6). *Significantly different from saline vehicle control (*p* < 0.05).

**Figure 3:**
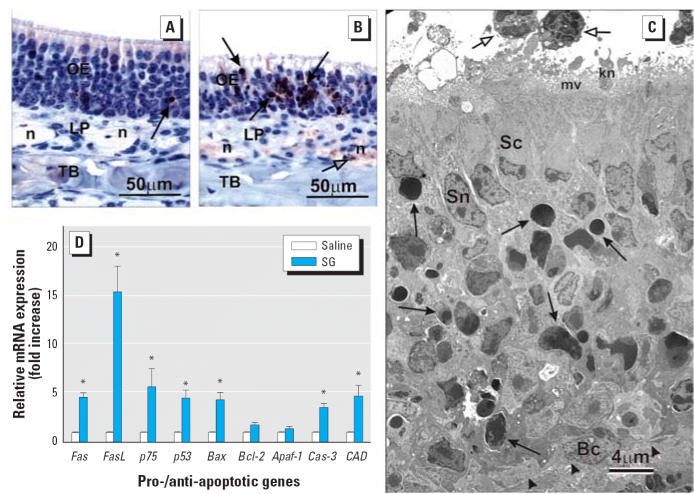
Apoptosis of OSNs in OE 24 hr after initial exposure to SG. Abbreviations: 2E, ethmoid turbinate 2; Bc, basal cell; LP, lamina propria; kn, dendritic knob of OSN; mv, microvilli; n, axons of OSNs; Sc, sustentacular cell cytoplasm; Sn, sustentacular cell nucleus; TB, turbinate bone. (*A* and *B*) Immunohistochemical detection of activated caspase-3 (brownish red) in OE lining the medial aspect of 2E in T3 and olfactory nerve bundles in the underlying LP in mice treated with saline vehicle alone (*A*) or 500 μg/kg bw SG (*B*). The open arrow in (*B*) indicates the immunohistochemical staining of nerve bundles in LP; solid arrows indicate stained OSNs in OE. (*C*) Electron photomicrograph of SG-exposed (500 μg/kg bw) OE with numerous apoptotic bodies or shrunken OSNs with condensed or fragmented nuclei or marginated nuclear chromatin (solid arrows); open arrows indicate neutrophils and arrowheads indicate subepithelial basal lamina. (*D*) Relative mRNA expression of apoptosis-related genes in excised ethmoid turbinates 1 day PI with saline or 500 μg/kg bw SG; data are means ± SEs (*n* = 6). *Significantly different from the saline vehicle control (*p* < 0.05).

Dose–response analysis of SG-induced apoptotic lesions indicated that the no-effect level was 5 μg/kg bw (80 ng/mouse) and the lowest effect level was 25 μg/kg bw (400 ng/mouse; Figure 2). SG-induced apoptosis was defined by condensation and shrinkage of individual epithelial cells; clumping, fragmentation, and margination of nuclear chromatin; and numerous widely scattered cellular fragments (apoptotic bodies) (Figure 3A–C). Apoptosis was restricted to OSNs whose cell bodies and nuclei reside in the middle nuclear layers of the OE below the distinct apical row of sustentacular (support) cell nuclei and above the basal cell nuclei near the basal lamina. Shrunken OSNs and associated apoptotic bodies expressed the inducible apoptotic marker protein caspase-3 at 1 day PI (Figure 3B). Prominent anti-caspase-3 staining was also present in olfactory nerve bundles (axons of OSNs) in the underlying lamina propria of the mice instilled with SG. Real-time PCR analysis of microdissected OE-lined ethmoid turbinates from SG-treated mice demonstrated a marked up-regulation of the proapoptotic genes *Fas*, *FasL*, *p75NGFR*, *p53*, *Bax*, caspase-3, and *CAD*, whereas expression of *Apaf-1* and the anti-apoptotic gene *Bcl-2* was unchanged (Figure 3D).

### SG-mediated OE atrophy

**Figure 4:**
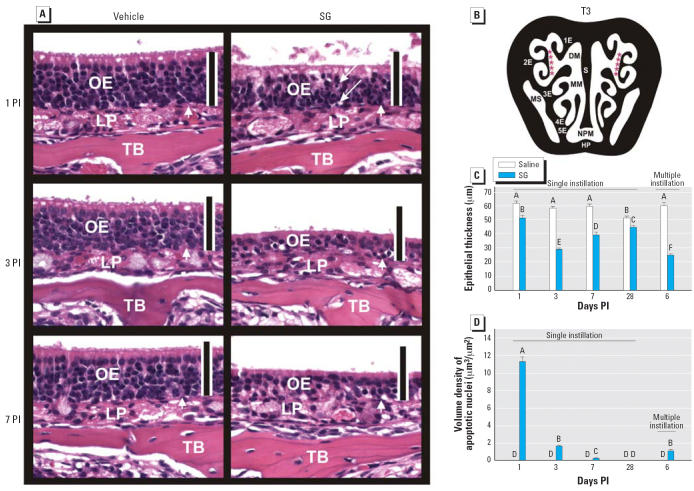
Time-dependent OE atrophy and OSN loss. Abbreviations: DM, dorsal medial meatus (airway); HP, hard palate; LP, lamina propria; MM, middle meatus; MS, maxillary sinus; NPM, nasopharyngeal meatus; S, septum; TB, turbinate bone. (*A*) OE at 1, 3, and 7 days PI in mice treated with saline vehicle alone or SG (500 μg/kg bw). Tissues were stained with H&E; bars = 50 μm. Arrows indicate apoptotic nuclei. (*B*) Intranasal site (2E, red asterisks) used for morphometry. Morphometric analysis of epithelial thickness (atrophy) (*C*) and volume density of apoptotic nuclei (*D*) in OE at 1, 3, 7, or 28 days after a single instillation with saline or SG 500 μg/kg bw or 6 days after the start of five daily instillations with saline or SG (100 μg/kg bw). Bars represent group means ± SE (*n* = 6). Bars labeled with different letters are significantly different (*p* < 0.05).

Concurrent with SG-induced OSN apoptosis, OE atrophy was detectable at 1 day PI (Figure 4A). SG-induced OE lesions were similarly apparent at 3 and 7 days PI along with increased atrophy. Morphometry of OE lining 2E (Figure 4B) confirmed that greater atrophy occurred in mice sacrificed 3 and 7 days PI than in mice sacrificed 1 day PI, with an approximately 50% reduction in epithelial thickness compared with control mice (Figure 4C). Restoration of the normal thickness of the OE compared with that of control was still not complete at 28 days PI (Figure 4C). Compared with SG-exposed OE at 1 day PI, volume density of apoptotic nuclei within the OE was remarkably reduced at 3 days PI and absent at 7 and 28 days PI (Figure 4D). Exposure to a lower daily dose of SG (100 μg/kg bw or 1.6 μg/mouse) for 5 consecutive days resulted in similar atrophy and apoptosis of OE compared with those alterations caused by the single 500 μg/kg bw SG (Figure 4C,D), suggesting that, in the short term, these effects were cumulative.

### Role of trichothecene structure in OE atrophy

**Figure 5:**
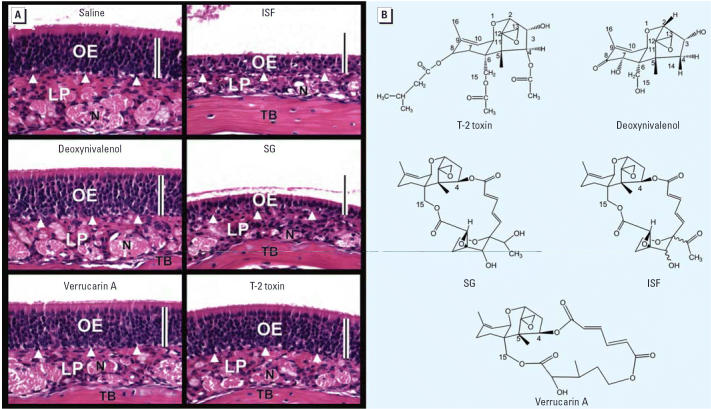
Comparative effects of trichothecenes on the induction of atrophy in OE. Abbreviations: LP, lamina propria; N, olfactory nerve; TB, turbinate bone. (*A*) Light photomicrographs of H&E-stained OE lining 2E in T3 from mice 3 days PI with saline vehicle alone, deoxynivalenol (10 mg/kg bw), verrucarin A (500 μg/kg bw), ISF (500 μg/kg bw), SG (500 μg/kg bw), or T-2 toxin (1 mg/kg bw); bars = 50 μm. (*B*) Chemical structures of tested trichothecenes.

The nasal effects of trichothecenes not associated with *Stachybotrys* were also assessed. Mice intranasally exposed to deoxynivalenol, T-2, and verrucarin A, which are type A, type B, and macrocyclic trichothecenes, respectively, using doses of equivalent to one-third to one-fifth of LD_50_ values (doses lethal in 50% of test animals) (Ueno 1984). These trichothecenes had no effect on OE compared with the *Stachybotrys* mycotoxins SG and ISF, which exhibited robust toxicity (Figure 5A). Thus, the trichothecene nucleus with characteristic 12–13 epoxide found in trichothecenes was insufficient to induce OE atrophy in mice; rather, the effect appeared to be dependent on satratoxin structure (Figure 5B).

### Selective apoptosis induction in OSNs

**Figure 6:**
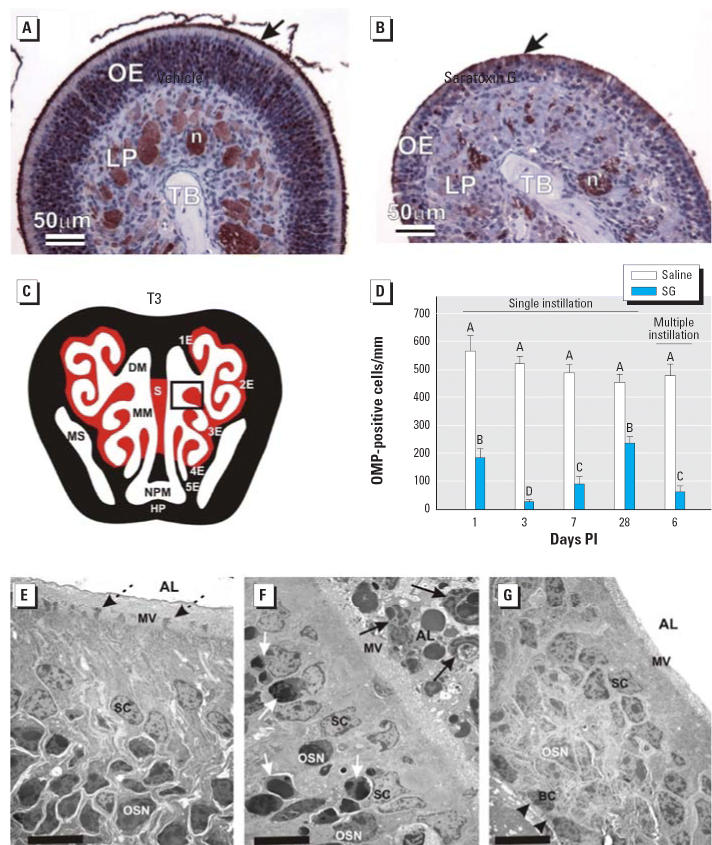
SG exposure depletes OSNs in OE. Abbreviations: AL, airway lumen; BC, basal cells; DM, dorsal medial meatus (airway); HP, hard palate; LP, lamina propria; MM, middle meatus; MS, maxillary sinus; MV, microvillar apical surface; S, septum; SC, sustentacular cells; TB, turbinate bone. (*A*,*B*) Light photomicrographs of OE lining 3E in T3 nasal section (illustrated in *C*) from mice 1 day PI with (*A*) saline vehicle alone or (*B*) SG (500 μg/kg bw). Sections were immunohistochemically stained with anti-OMP antibody and counter-stained with hematoxylin; immunoreactivity for OMP (marker for mature OSNs) is identified by the brownish red chromagen in OE and olfactory neurons (n) in the LP. Arrows in (*A*) and (*B*) indicate OMP-stained cilia along the airway surface; (*C*,*D*) Results of morphometric analyses of 3E (box) in T3 nasal section (*C*) are graphically illustrated in (*D*). Bars represent group means ± SEs. Bars labeled with different letters are significantly different (*p* < 0.05). (*E*–*G*) Three transmission electron photomicrographs of the apical third of the OE from a mouse 1 day after saline vehicle alone instillation (*E*) or 1 day after a single SG instillation (*F*), and the full thickness of remaining atrophic OE from a SG-exposed mouse 3 days after a single intranasal instillation (*G*); bars = 10 μm. Dotted arrows in (*E*) indicate normal dendritic knobs of OSNs, but there is a loss of these dendritic portions of OSNs in (*F* and *G*). In (*F*), white arrows indicate apoptotic OSNs and black arrows indicate luminal cellular debris and phagocytizing neutrophils in the nasal AL. Arrowheads in (*G*) indicate subepithelial basal lamina; only a few OSNs remain in the atrophic OE in (*G*).

OMP, a specific peptide found only in mature OSNs (Kream and Margolis 1984), was markedly reduced in the OE of SG-instilled mice compared with saline-instilled control mice (Figure 6A–C). Morphometric analysis revealed a 67, 94, and 81% loss in OSNs per millimeter of OE in the nasal mucosa lining the dorsolateral meatus at 1, 3, and 7 days PI, respectively, compared with the identical region in vehicle-instilled mice (Figure 6D). Consistent with partial recovery of OE thickness at 28 days PI (Figure 4C), there was also incomplete restoration of the numbers of OMP-positive OSNs (Figure 6D) in the nasal mucosa lining the dorsolateral meatuses of OSNs and OE thickness. OSNs, unlike neurons in other parts of the body, have the ability to regenerate from OE basal cells and restore their synaptic connections in the OB (Graziadei and Graziadei 1978). Dosing with five consecutive daily SG instillations (100 μg/kg bw) resulted in an 87% loss of OSNs, again suggesting that neuronal effects were cumulative. At the ultrastructural level, SG-induced atrophic OE had a conspicuous loss of nuclear and cytoplasmic profiles of OSNs and their ciliated dendritic knobs that contain the animal’s odorant receptors and normally project above the microvillar apical surfaces of the sustentacular cells (Figure 6E–G). Consistent with OSN loss, both ultrastructural and immunohistochemical examination demonstrated a marked reduction of the normally dense mat of cilia projecting from the dendritic knobs and lining the surface of the nasal airway lumen (Figure 6B,G).

**Figure 7:**
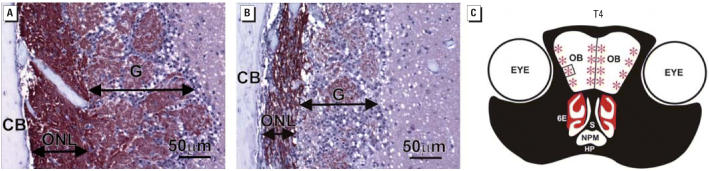
Depletion of OSNs in OBs. Abbreviations: CB, cranial bone; G, glomerular layer; HP, hard palate; ONL, olfactory nerve layer; NPM, nasopharyngeal meatus; S, nasal septum. (*A* and *B*) Immunohistochemical detection of OMP (brownish red) in ONL and G layer in the OB of mice 7 days PI with (*A*) saline vehicle alone or (*B*) SG (500 μg/kg bw). (*C*) T4 nasal section containing OB and sites of ONL and G layer atrophy due to SG exposure (red asterisks). Square in (C) represents the location in OB of (*A*) and (*B*).

Concurrent with the initial loss of OMP-positive OSNs, there was also noticeable atrophy of the OMP-positive olfactory nerve bundles located in the lamina propria underlying the atrophic OE (Figure 6B). This corresponded to marked bilateral atrophy of the olfactory nerve layer and adjacent glomerular layer comprising the outer two tissue layers of the OB in SG-instilled mice (Figure 7A,B). Loss of OMP-stained olfactory nerves was most marked in the lateral and medial aspects of each of the OBs (Figure 7C).

### Inflammatory gene up-regulation and neutrophil infiltration in OE and OB

**Figure 8:**
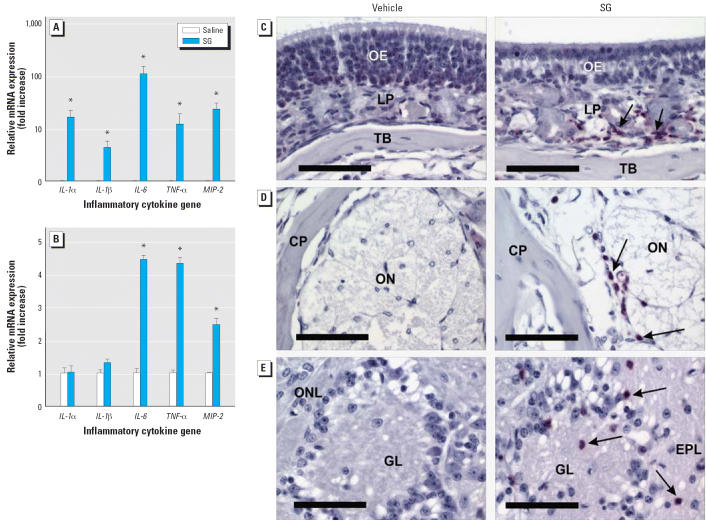
Acute inflammatory responses in mouse nasal passage (rhinitis) and OB (mild focal encephalitis). Abbreviations: CP, cribiform plate; EPL, external plexiform layer; ONL, olfactory nerve layer; and GL, glomerular layer; LP, lamina propria; ON, olfactory nerve bundles; TB, turbinate bone. (*A*, *B*) Real-time PCR measurements of proinflammatory gene mRNAs in ethmoid turbinates (*A*) and OB (*B*) of mice 1 day PI with saline vehicle or SG (500 μg/kg bw); bars represent group means ± SE (*n* = 6). (*C*–*E*) Immunohistochemical detection of neutrophils (red stained cells and indicated by arrows) in OE (*C*), ON passing through the CP from nose to OB (*D*), and the ONL and GL in the OB (*E*) from mice 7 days PI with saline vehicle alone or SG (500 μg/kg bw); bars = 50 μm. *Significantly different from control group (*p* < 0.05).

At 1 day PI, SG also induced conspicuous accumulations of exfoliated and degenerating cellular debris from the dendritic portions of the apoptotic OSNs in the nasal airways along the luminal surfaces of the atrophying OE. With secondary degeneration of these exfoliated dendritic fragments, there was accompanying infiltration of numerous phagocytic cells consisting mainly of polymorphonuclear leukocytes (neutrophils) and only occasional mononuclear cells (monocytes and macrophages). Many of the luminal neutrophils had engulfed apoptotic cellular fragments (Figures 3C, 6F). Phagocytosis of apoptotic bodies by sustentacular cells within the OE was also evident by ultrastructural examination. Lesser numbers of infiltrating neutrophils were also widely scattered in lamina propria of the SG-altered olfactory mucosa. Consistent with leukocyte influx was a markedly increased expression of mRNAs for the cytokines TNF-α, IL-1α, IL-1β, and IL-6 as well as the chemokine MIP-2 associated with acute inflammatory cell infiltration (Figure 8A). Elevated *MIP-2*, *TNF-*α, and *IL-*6 mRNA expression was also observed in the OB (Figure 8B).

**Figure 9:**
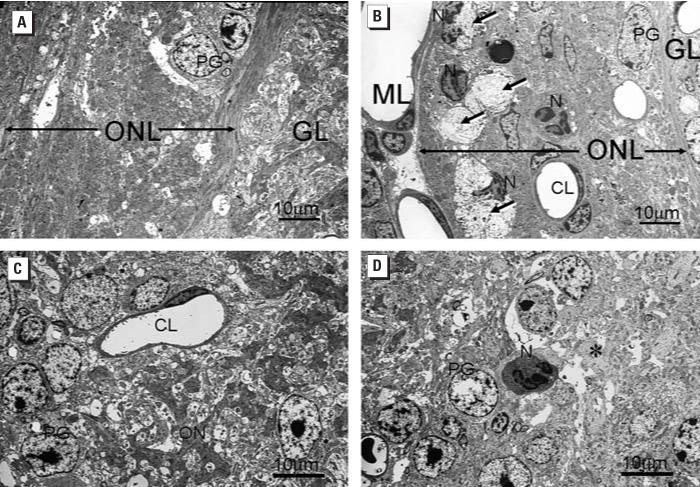
Ultrastructural alterations in ONL and GL of OB 7 days PI with saline vehicle alone (*A*, *C*) or SG (500 μg/kg bw) (*B*, *D*). Abbreviations: CL, capillary lumen; GL, glomerular layer; ML, meningeal layer; N, neutrophil; ON, olfactory nerves; ONL, olfactory nerve layer; PG, periglomerular cell. (*A*, *B*) Focal areas of axonal degeneration (long arrows) present in the markedly atrophic ONL in the SG-treated mouse (*B*) but not the vehicle-treated control mouse (*A*). In (*B*), infiltrating Ns with segmented nuclei are closely associated with areas of degeneration (arrows). (*C*, *D*) Dense staining axons of ON in the GL of the vehicle control mouse (*C*) are absent in the GL of the SG-exposed mouse (asterisk in *D*). An N is also present in the SG-exposed mouse but not in the control mouse.

Slightly decreased severity of SG-induced neutrophilic rhinitis corresponded with time-dependent disappearance of epithelial apoptosis and development of epithelial atrophy. A mild-to-moderate influx of neutrophils persisted in the lamina propria of the affected nasal mucosa underlying the atrophic OE even at 7 days PI (Figure 8C). Remarkably, there were mild, widely scattered infiltrations of neutrophils in the remaining olfactory nerve bundles penetrating the bony cribiform plate that separates the nasal cavity and OB of the brain (Figure 8D). These infiltrations extended bilaterally into the atrophic olfactory nerve and glomerular layers of the OB at 7 days PI (Figure 8E). Ultrastructural examination revealed that the infiltrating neutrophils were closely associated with focal areas of degeneration and loss of OSN axons in both of these outer layers of the OB (Figure 9A,B). A few isolated neutrophils were even detected in the deeper external plexiform layer, although no neuronal damage was evident in this or other areas of the OB of SG-exposed mice.

## Discussion

**Figure 10:**
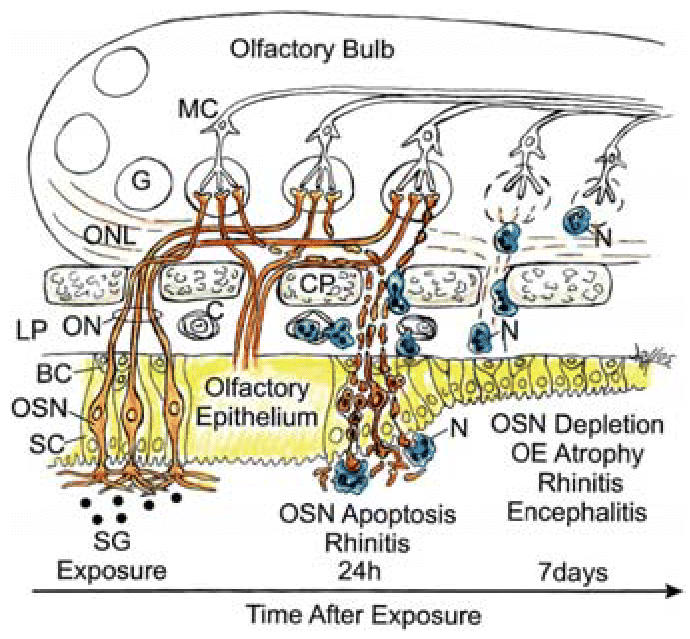
Diagrammatic representation of SG-induced pathology in OE and OB with time after exposure. Abbreviations: BC, basal cells; C, capillary in LP; CP, cribiform plate; G, glomerular; LP, lamina propria; MC, mitral cell; N, neutrophil; ON, olfactory nerve; ONL, olfactory nerve layer; SC, sustentacular cells. OE is shown in yellow, normal OSN in orange, and neutrophils phagocytosing apoptotic OSNs in blue.

The causes of damp-building syndrome are likely to be multifactorial and involve toxic, inflammatory, and allergic responses to microbes and their products; however, the underlying mechanisms, relative contributions of individual organisms, and potential for interactions remain poorly understood (IOM 2004). Although exposure to either *S. chartarum* spores or associated satratoxins has been previously shown to initiate acute inflammatory responses in the rodent lung (Yike and Dearborn 2004), our observations that very low doses of SG are directly toxic to OSNs and initiate an inflammatory response in the nose (rhinitis) that extends into the brain (mild focal encephalitis) (Figure 10) are heretofore unreported. These findings raise significant new questions about hazards associated with indoor exposure to this fungus in water-damaged buildings. Several aerosol studies have demonstrated that there is substantial deposition (> 50%) of either very large (> 5 μm in particle diameter; e.g., a fungal spore) and very small particles (< 10 nm in diameter; nanoparticles) in the nasal airways of humans and laboratory animals when inhaled through the nose (Cheng et al. 1990, 1991, 1996; Yeh et al. 1997). Therefore, it is very likely that the nasal airways will filter out the inhaled spores or extremely small fragments emitting from the mold, preventing deposition in the lower respiratory tract, including the lungs.

Like other epithelial cells in the body, but unlike most neuronal cell populations in the mammalian nervous system, OSNs undergo apoptosis and genesis throughout the life of the animal as part of the normal turnover of mature OE. OSNs are unique in that they have relatively short life spans compared with other neurons and are continuously being replaced through basal cell proliferation and differentiation (neuronal regeneration) (Graziadei and Graziadei 1978). Most OSNs live for 30–40 days, but some cells have life spans of 3 months or even longer. OSNs of laboratory animals may be induced to die *in vivo* by experimentally manipulative methods that include olfactory bulbectomy, transection of the olfactory nerve at the cribiform plate, and intranasal exposure to chemicals known to be toxic to the OE, such as zinc sulfate and methyl bromide (Cowan and Roskams 2002). Exposures to most olfactory chemical toxins result in necrosis (oncosis) of the OSNs along with other epithelial cells in the OE, unlike the selective cell death of OSNs by apoptosis observed in the present study. Recently, however, exposure of mice to some chemotherapeutic agents, such as vincristine, was found to induce marked apoptosis of OSNs with subsequent OE atrophy that resembles SG-induced lesions described herein but without obvious nasal inflammation (Kai et al. 2004). In contrast to our study, mice in these previous studies were given the chemical agents systemically and at much higher doses relative to body weight (milligrams per kilogram vs. micrograms per kilogram).

SG might drive both extrinsic (death receptor–mediated) and intrinsic (mitochondrial-mediated) apoptotic pathways in OSNs. The trichothecenes induce gene expression and apoptosis via a ribotoxic stress response that involves MAPKs (Shifrin and Anderson 1999; Yang et al. 2000) and is mediated upstream by double-stranded RNA–activated protein kinase (Zhou et al. 2003) and Src-family kinases (Zhou et al. 2005b). Notably, SG-induced genes that have previously been associated with death receptor–mediated OSN apoptosis include *TNF-*α, *Fas*, *FasL*, and *p75NGFR* (Cowan and Roskams 2002), as well as the downstream apoptotic genes *p53* (Huang et al. 1995), *Bax* (Ge et al. 2002), and caspase-3 (Cowan and Roskams 2004). Relative to the intrinsic pathway, trichothecene deoxynivalenol induces p38-mediated mitochondrial-dependent caspase-3 activation and apoptosis in cloned macrophages (Zhou et al. 2005a). Furthermore, satratoxin H–induced caspase-3 activation and apoptosis in the PC12 neural cell model have recently been reported to be both p38 and JNK dependent (Nusuetrong et al. 2005).

It is unclear why SG specifically targeted OSNs when nasal respiratory epithelium and other cell types in the OE were unaffected. OSN sensitivity to SG might relate to longer regional exposure to epithelial cells in OE compared with the exposure to cells in respiratory epithelium. This is possibly due to a much slower rate of mucociliary clearance of inhaled agents from OE-lined ethmoid turbinates, which are covered by immotile cilia, compared with other parts of the nasal cavity that are lined by respiratory epithelium containing motile cilia with high ciliary beat frequencies. This latter movement generates rapid regional flows of mucus out of the nasal cavity and through the nasopharynx into the upper digestive tract (Morgan et al. 1984). A slower rate of intranasal SG clearance from OE compared with respiratory epithelium may also be due to differences in other factors known to affect the clearance of chemicals from the nasal airway, such as mucosal metabolism or blood flow.

Alternatively, based on our observations that satratoxin-induced OE atrophy is highly dependent on chemical structure (Figure 5) and that one region of the nasal cavity lined by OE (dorsomedial meatus) was consistently spared from toxicant-induced injury (Figure 1), it is tempting to speculate that these trichothecenes or as yet unidentified metabolites bind to specific OSN receptors, thus facilitating uptake and resultant toxicity. In support of this contention, populations of distinct odorant receptors can be divided into four specific topographical regions of the OE, one of which lines the dorsomedial meatus (Ressler et al. 1993).

Taken together, our observations that the OE and OB are targets of SG and ISF should be a critical consideration in future studies of damp-building–related illnesses and the potential etiologic role of *S. chartarum*. The profile of induced cytokines and *MIP-2* is likely to contribute to OSN apoptosis as well as accompanying rhinitis and mild focal encephalitis observed in the present study. In the future, it will be necessary to ascertain the dose–response effects and latency of recovery in nasal tissue after chronic exposure to satratoxins alone, as well as the contributions of spore matrix, or coexposures to other indoor air contaminants such as endotoxin. Particularly intriguing will be understanding the basis for OSN specificity and the role of toxin metabolism. Of further critical importance will be the extent to which toxicant-induced inflammation and neuronal injury occur in other parts of the brain along the olfactory pathway and whether this contributes to neurocognitive dysfunction. Ultimately, all such information must be framed against accurate quantitative assessments of human exposure to satratoxins using both state-of-the-art sampling and analytical methods and relevant biomarkers.
